# Multi-Response Optimization of WEDM Process Parameters for Machining of Superelastic Nitinol Shape-Memory Alloy Using a Heat-Transfer Search Algorithm

**DOI:** 10.3390/ma12081277

**Published:** 2019-04-18

**Authors:** Rakesh Chaudhari, Jay J. Vora, S. S. Mani Prabu, I. A. Palani, Vivek K. Patel, D. M. Parikh, Luis Norberto López de Lacalle

**Affiliations:** 1 Department of Mechanical engineering, School of Technology, Pandit Deendayal Petroleum University, Raisan, Gandhinagar 382007, India; chaudharirakesh5@gmail.com (R.C.); viveksaparia@gmail.com (V.K.P.); 2 Metallurgy Engineering and Materials Science, Indian Institute of Technology Indore, Indore 453552, India; maniprabhu.sakthivel@gmail.com (S.S.M.P.); palaniia@iiti.ac.in (I.A.P.); 3 Discipline of Mechanical Engineering, Indian Institute of Technology Indore, Indore 453552, India; 4 Department of Industrial engineering, School of Technology, Pandit Deendayal Petroleum University, Raisan, Gandhinagar 382007, India; dm.parikh@sot.pdpu.ac.in; 5 Department of Mechanical Engineering. University of the Basque Country, Escuela Superior de Ingenieros Alameda de Urquijo s/n., 48013 Bilbao, Spain; norberto.lzlacalle@ehu.eus

**Keywords:** shape memory alloy, superelastic nitinol, WEDM, heat transfer search algorithm, DSC test, shape memory effect

## Abstract

Nitinol, a shape-memory alloy (SMA), is gaining popularity for use in various applications. Machining of these SMAs poses a challenge during conventional machining. Henceforth, in the current study, the wire-electric discharge process has been attempted to machine nickel-titanium (Ni55.8Ti) super-elastic SMA. Furthermore, to render the process viable for industry, a systematic approach comprising response surface methodology (RSM) and a heat-transfer search (HTS) algorithm has been strategized for optimization of process parameters. Pulse-on time, pulse-off time and current were considered as input process parameters, whereas material removal rate (MRR), surface roughness, and micro-hardness were considered as output responses. Residual plots were generated to check the robustness of analysis of variance (ANOVA) results and generated mathematical models. A multi-objective HTS algorithm was executed for generating 2-D and 3-D Pareto optimal points indicating the non-dominant feasible solutions. The proposed combined approach proved to be highly effective in predicting and optimizing the wire electrical discharge machining (WEDM) process parameters. Validation trials were carried out and the error between measured and predicted values was negligible. To ensure the existence of a shape-memory effect even after machining, a differential scanning calorimetry (DSC) test was carried out. The optimized parameters were found to machine the alloy appropriately with the intact shape memory effect.

## 1. Introduction

Nitinol is a nickel-titanium alloy which exhibits outstanding properties such as shape memory, superelasticity, and biocompatibility. A shape-memory alloys exhibit in numerous forms. One of the shape memory alloys which possesses superelasticity and biocompatibility is nickel–titanium alloy. Nickel-titanium alloys are also commonly referred to as nitinol in honour of its discovery at the Naval Ordnance Laboratory (NOL) and are a preferred material specifically for biomedical applications due to their high corrosion and wear resistance, pseudoelasticity and biocompatibility [1,2,3]. Shape-memory alloys (SMAs) when heated above the transition temperature recover their previous deformed shape. Recoverable elastic deformation of superelastic nitinol which is considered to be a new generation smart material is significantly larger than the conventional materials [1]. Phase transformations of SMAs are exhibited in the austenite and martensite phase. Austenite is stable at a higher temperature and has a body center cubic structure whereas martensite is stable at a lower temperature and has a monoclinic crystal structure. SMAs exhibit two effects, super-elastic effect and shape-memory effect. If the austenite finish temperature is below room temperature then particular material is said to produce a super-elastic effect whereas if austenite finish temperature is above room temperature then the material exhibits a shape-memory effect [1]. Due to large deformation recovery and higher wok density, NiTi alloy also finds its application in various areas like aerospace, sensors, robotics, actuators, automotive, structural elements etc. [4,5]. However, high ductility, typical stress-strain behavior, and superelasticity make nitinol difficult to cut using conventional machining processes due to short tool life, a reduced quality of the workpiece, and burr formation [6,7,8,9]. Past studies of SMAs with conventional machining reports poor chip breaking, high tool wear, poor surface quality, low-dimensional accuracy and most importantly retaining the shape memory effect after machining [9,10,11]. Thus, SMAs and most preferably nitinol are best machined through non-conventional machining techniques.

Wire electrical discharge machining (WEDM) is one of the non-conventional machining processes which is a non-contact type process, where the material is removed with the help of high frequency sparks generated between the tool and workpiece [12]. Due to the absence of physical contact of tool and workpiece, this process can be used for any type of material regardless of their hardness, provided the material is electrically conductive [13,14]. This process is used to obtain intricate and complex shape geometries with close tolerances [15]. However, WEDM process involves a high number of input process parameters which needs to be set to their optimal level for achieving the required geometry with close co-relationship between multiple output responses along with the metallurgical and mechanical properties. In accordance with this, most of the past research studies are pivoted to parametric optimization of the WEDM process using different optimization techniques.

Bisaria and Shandilya [16] used the WEDM process for the machining of Ni55.95Ti44.05 SMA. The influence of input parameters such as pulse on time (T_on_), pulse off time (T_off_), spark gap voltage (SV), wire tension (WT), wire feed rate (WF) has been studied on material removal rate (MRR), surface roughness (SR) and surface characteristics of material. They found that MRR and SR values increase significantly with the increase in T_on_ whereas MRR and SR values decrease with an increase in SV and T_off_. Micro-cracks, craters, and debris were observed on the machined surface. The defect could be eliminated by more precise controlling of WEDM process parameters. Sharma et al. [17] conducted the parametric optimization of Ni40Ti60 alloy using the WEDM process. MRR, SR and the dimensional shift have been considered as an output response variables under the influence of input variables such as T_on_, T_off_, peak current (IP) and servo voltage (SV). Output responses were optimized using desirability approach for SMA alloy and close correlation between predicted and experimented values were obtained. T_on_ of 124 µs, T_off_ of 25 µs, SV of 30 V, and IP of 110 mu was the obtained optimal parameter setting for multi-response optimization with desirability value of 0.708. In another study by Soni et al. [18], WEDM machining of Ti50Ni40CO10 SMA has been explored. The final result revealed formation of microcracks can be avoided and recast layer thickness can be reduced by setting pulse on time lower than 125 µs and servo voltage larger than 20 V. Majumder and Maity [19] conducted a similar study wherein microhardness (MH) and SR were considered as output response variables and they are optimized with the help of a fuzzy technique for the SMA alloy Ni55Ti45. T_on_ was identified as the main significant input process parameter as compared to other input variables. Manjaiah et al. [20] used a L_27_ orthogonal array to perform the experiment and optimized output responses of MRR and SR during the machining of SMA. The study highlighted the significant effect of T_on_ and T_off_ and SV for the maximization of MRR and minimization of SR under the influence of brass wire and zinc-coated brass wire. B. Jabbaripour et al. [15] states that the electrical discharge machining (EDM) process is suitable for machining titanium alloys. They used Ti6Al4V to investigate various output performance characteristics. Increase in T_on_ resulted in increase in MRR. In another study by Ramamurthy et al. [21], machining of Ti6Al4V alloy was conducted using the EDM process by using Taguchi L9 orthogonal array. They observed that T_off_ has more influent nature on the output performances of machining characteristics because the T_off_ influences the discharge energy in WEDM.

From this preliminary survey, it can be summarized that the optimization of process parameters of machining of SMA alloys has been carried out primarily for MRR, SR, and MH. However, the effect of these optimized parameters has not been explored on the shape memory effect of the machined surface. In addition to this, the majority of the studies optimized an individual response rather than simultaneous optimization.

WEDM is a multi-input multi-output process encompassing complex dependencies on individual parameters as well as their interactions [21,22]. Generally, wherever multiple objectives are considered, several conflicting situations arise wherein there is a requirement is to settle at a tradeoff between these multiple responses. This tradeoff is best presented by optimal Pareto points generated using advanced evolutionary optimization techniques. This method gives the advantage by finding the solution near global optimum with reduced time and computational efforts by generating optimal Pareto points. Different algorithms such as particle swarm optimization, genetic algorithms, ant colony optimization etc. which are nature-based optimization ideologies have been widely experimented with for different optimization problems including WEDM [23,24,25,26]. However, these evolutionary algorithms function under a set of assigned values which is algorithm-specific. Precise control of these parameters will dictate the performance of those algorithms for optimization. To overcome this challenge, new algorithms were developed by researchers wherein tuning of those algorithm-specific parameters was not required. The heat-transfer search (HTS) algorithm is one such technique with the major advantage being proper balancing between exploration and exploitation. The proper balancing is incorporated by introducing six different search mechanisms in algorithm. The different search mechanism is generated by number of generations of the algorithm. In addition to that HTS is easy to implement and can find the global optimal solution for complex problems. These noticeable advantages of the HTS algorithm are observed at the cost of computation time. HTS has been successfully applied to different benchmarking problems pertaining to different fields [27,28,29,30,31]. However, to the best of the authors’ knowledge, no study has been reported on the application of HTS for manufacturing problems.

Pursuant to a detailed review of an available research article, it can be recognized that T_on_, T_off_ and the current are the three most notable input process parameters while MRR, SR, and MH as the output response variables. The prime requirement after machining of SMA is its shape-memory effect. Shape-memory effect has been correlated to MH [32]. The differential scanning calorimetry (DSC) test is one of the techniques to ensure shape memory effect. Along with this, either single or multi-objective optimizations with limited consideration to actual industrial requirements are targeted in past published studies. However, to the best of the author’s knowledge, generated Pareto curves are targeted only for two responses for the study of SMAs using the WEDM process [33]. Thus, the present study addresses an evident research gap by using pareto curves incorporating three simultaneous responses generated using a novel HTS algorithm.

In the current study, a detailed study on WEDM of superelastic Ni55.8Ti alloy has been carried out and the aforementioned research gap has been fulfilled. The Box-Behnken technique of response surface methodology (RSM) has been used to conduct the experiments and three important output responses were recorded. Furthermore, for each of those output responses, mathematical models were generated and tested by analysis of variance (ANOVA) and residual plot analysis was carried out to verify ANOVA results. Using an advanced parameter less evolutionary algorithm known as HTS, multiple case studies including real-time manufacturing scenario along with simultaneous optimization of the output responses have been carried out. 3D and 2D Pareto curves have been generated with the help of multi-objective HTS algorithm which displays different non-dominant optimal points. Confirmation trails have been conducted to compare the results of predicted and measured responses. To check the shape memory effect, the DSC test has been carried out on the machined surface obtained with optimized parameters during the validation test. The authors firmly believe the study would provide substantial input to the end users working of WEDM of superelastic SMAs.

## 2. Materials and Methods

**Figure 1:**
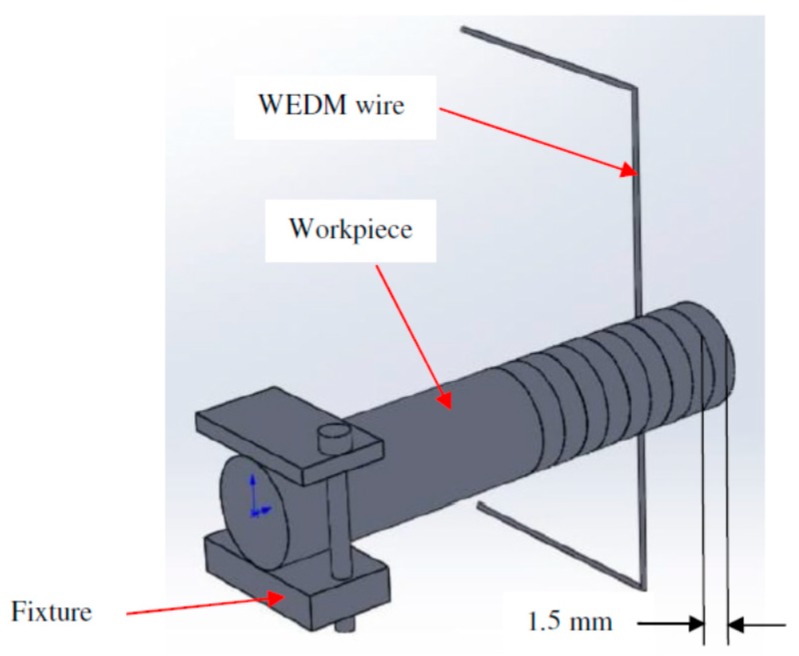
Schematic representation of wire electrical discharge machining (WEDM) process.

In the present study, superelastic shape memory nitinol (Ni5.8Ti) is selected as a workpiece material. In all the experimental trials, slices of 1.5 mm were cut from the rod of 8 mm diameter with the WEDM process. A schematic of the WEDM process for the present study is shown in Figure 1. Chemical composition (wt.%) of Ni55.8Ti is as shown in Table 1. As received SMA has a tensile strength of 750 MPa with 11% elongation. Experiments were conducted on a Concord WEDM machine DK7732 (Concord Limited, Bangalore, India) with dielectric fluid. Table 2 shows the selected range of machining parameters such as T_on_, T_off,_ and current along with 3 different levels which have been selected on the basis of existing literature, machining capability and their influence on selected output response parameters. Ni55.8Ti was used as a workpiece (anode) and 0.18 mm diameter molybdenum wire was used as a tool electrode (cathode). Experiments (3 number of trials at each parameter setting) have been conducted following the Box-Behnken (BBD) technique of RSM as shown in Table 3. Response surface methodology has been used to minimize the number of trials which reduces the cost of material as well as reduces the machining time.

The material removal rate is calculated by the difference in weight of the sample before and after machining carried out per second. Equation (1) shows the method to calculate MRR in mm^3^/s.

(1) $$\mathrm{MRR}=\frac{\Delta W\times 1000}{\rho \times t}$$

where, ΔW = weight loss from the workpiece,

t = duration of the machining process in second,
ρ = 6.5 g/cm^3^ the density of the workpiece

**Figure 2:**
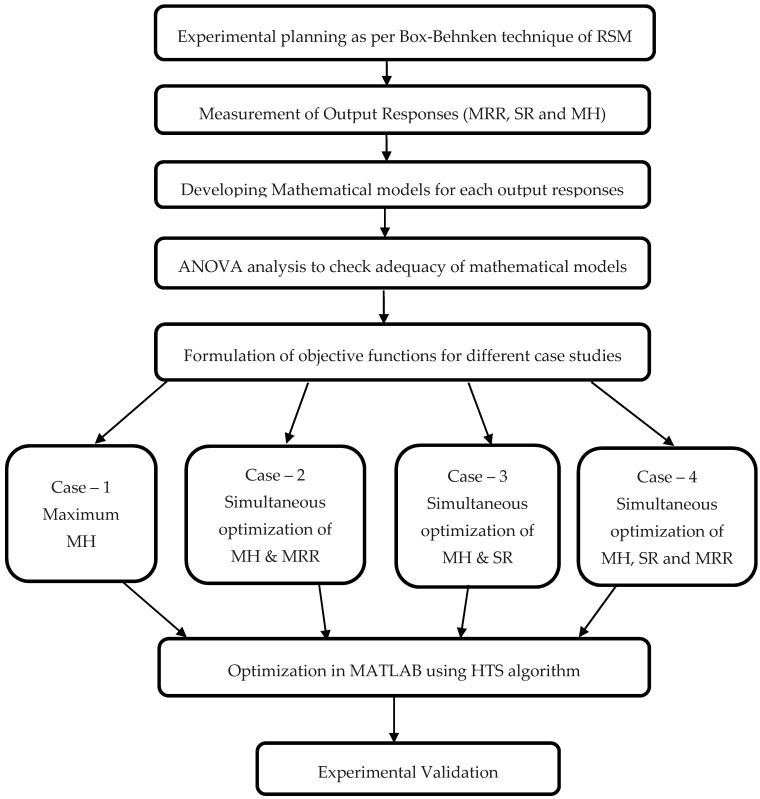
Proposed optimization route.

The Mitutoyo Surftest SJ-410 model (Mitutoyo ltd., New-Delhi, India) surface roughness tester was used to measure the surface roughness of the machined sample. The SR of each sample was measured at four different locations and the average value was taken as the output response. The cutoff length (λ_c_) was selected as 0.8 mm with the evaluation length of 7 mm. Ra values were recorded and analyzed to indicate the surface quality of the cut. A mirror finish was developed on the machined sample for the micro-hardness testing. A Vickers microhardness tester (MVH-S Auto Omintech, Pune, India) was used to calculate microhardness of the surface at 500 gf load at 10 s. The measured values of MRR, SR, and MH are analyzed and shown in Table 3 and the mathematical correlation was developed for each response. Further, the optimization route was followed as given in Figure 2. Validation of the shape-memory effect was conducted by the DSC technique. The DSC test was used to study the phase transformation behavior for both machined/unmachined surfaces and the results were compared. Using a Netzsch DSC 214 polyma machine (Netzsch, Selb, Germany), the DSC test was performed with a sample weight of 20 g at heating/cooling rate of 10 °C/min and a constant flow of nitrogen. The sample was place in a pan and a small spear hole was drilled on the top of the pan. This pan was kept within the machine for testing.

## 3. Results and Discussion

### 3.1. Generation of Mathematical Model

Table 3 shows the results obtained for MRR, SR, MH by using the design matrix of BBD. The maximum and minimum MRR values of 1.2309 mm^3^/s to 0.5414 mm^3^/s respectively were obtained. SR value ranging from 4.944 µs to 6.82 µs and MH ranging from 275.2 HV to 383.9 HV were achieved. It can be observed from Table 3 that a different set of output responses was obtained at a different input set of parameters. The output response values mentioned in Table 3 were analyzed using Minitab software (Version 17) to generate in terms of input variables as shown in Equations (2)–(4):

(2) $$\mathrm{MRR}=1.1400-0.0168\cdot A-0.0838\cdot B+0.5430\cdot C-0.0548\cdot C^{2}+0.0010\cdot A\cdot B$$

(3) $$\mathrm{SR}=0.2300-0.18000\cdot A+0.1974\cdot B+4.8290\cdot C+0.0040\cdot A^{2}-0.2540\cdot C^{2}-0.0569\cdot A\cdot C-0.0429\cdot B\cdot C$$

(4) $$\mathrm{MH}=521+10.9700\cdot A-39\cdot B-132.5000\cdot C-0.1575\cdot A^{2}+0.8400\cdot B^{2}+25.2300\cdot C^{2}+0.2770\cdot A\cdot B$$

where A is pulse on time, B is pulse off time and C is current.

Equations (2)–(4): are regression equations which primarily give the information on the effect of independent parameters (T_on_, T_off_ and Current) and their interactions on the dependent quality parameter (MRR, SR and MH). The ANOVA technique is used to investigate the significant and non-significant process parameters. The significance of input process parameter on the output response is indicated by F value and P value. The significance of process parameter on output response can be known from either higher F value or lower P value. The value of P must be lower than 0.05 to keep the particular process parameter significant for the 95% confidence level. The significance of T_on_ and T_off_ and current is shown in Table 4 on MRR, SR, and MH. T_off_ and current are found to be significant for the output response of MRR while T_off_ for SR and current for MH is considered to be the significant process parameters. Lack of fit was observed to be insignificant for all the responses which mean that the model is adequate for predicting the output responses under any combination of the process parameters considered in the range [34]. The 20% difference between R-squared and Adjusted (Adj) R-squared values means that the model is the best fit for selected responses [17]. For all the output responses considered in this study a difference of less than 20% was achieved.

**Figure 3:**
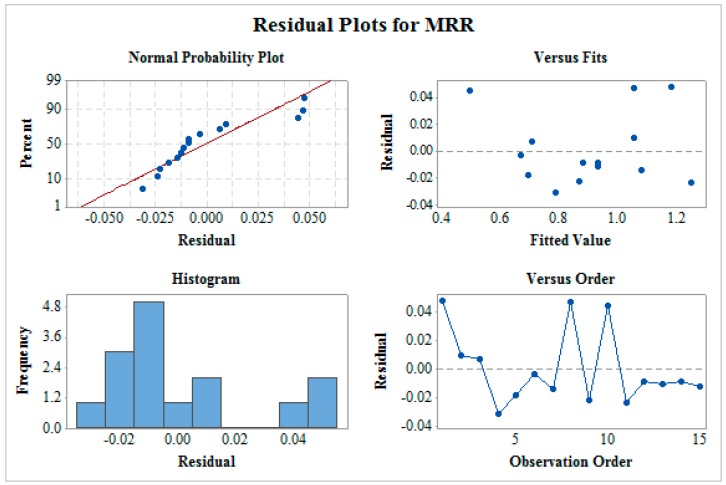
Residual plot for material removal rate (MRR).

**Figure 4:**
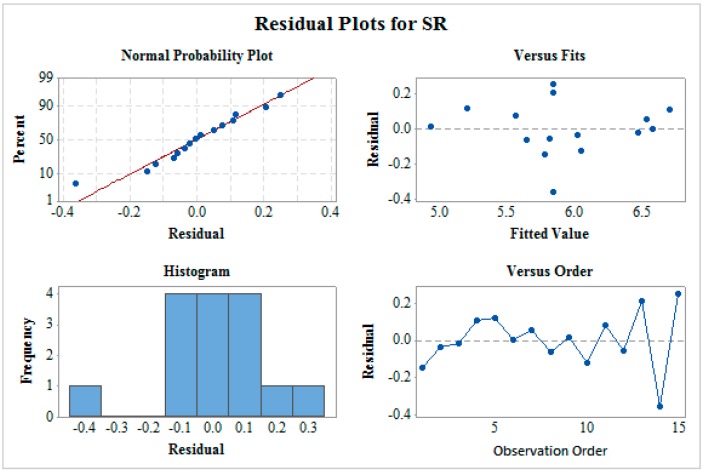
Residual plot for surface roughness (SR).

**Figure 5:**
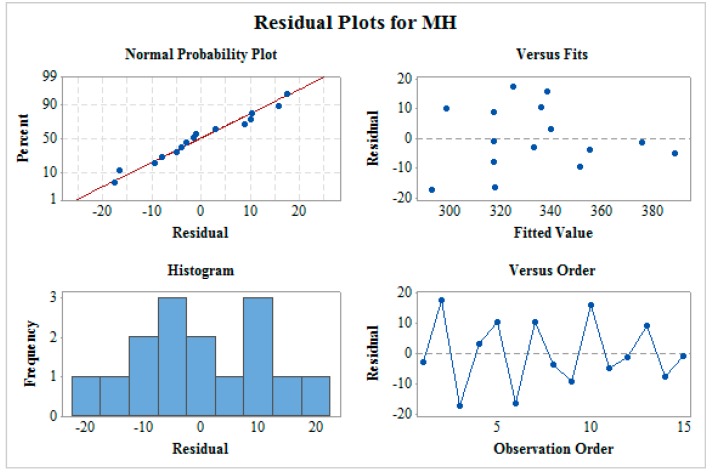
Residual plot for microhardness (MH).

Figure 3 shows the residual plot for MRR which includes normality plot, fitted versus predicted plot, histogram, and residual versus observation order plot. The normality plot shows that all the residuals are on the straight line which indicates the fitness of the proposed model. The random allocation of all the residuals on both the sides of the reference line can be seen from residual versus fitted plot. In a histogram plot, the bell-shaped curve is observed which support the normality of data. Furthermore, the residual versus observation order plot does not follow any pattern which is a mandatory case for significant ANOVA. This verifies that all the four tests confirms a satisfactory future outcome for the proposed model. In a similar way, residual plots as shown in Figure 4 and Figure 5 for SR and MH respectively verify ANOVA results.

**Figure 6:**
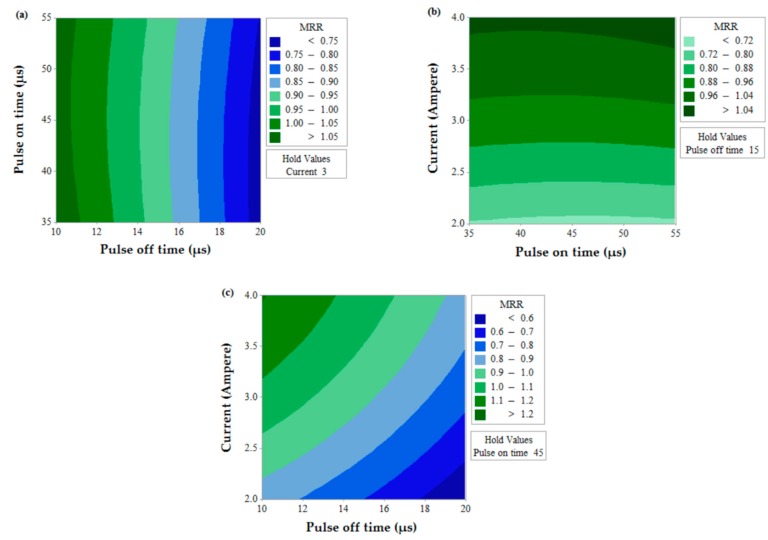
Contour plot for MRR.

**Figure 7:**
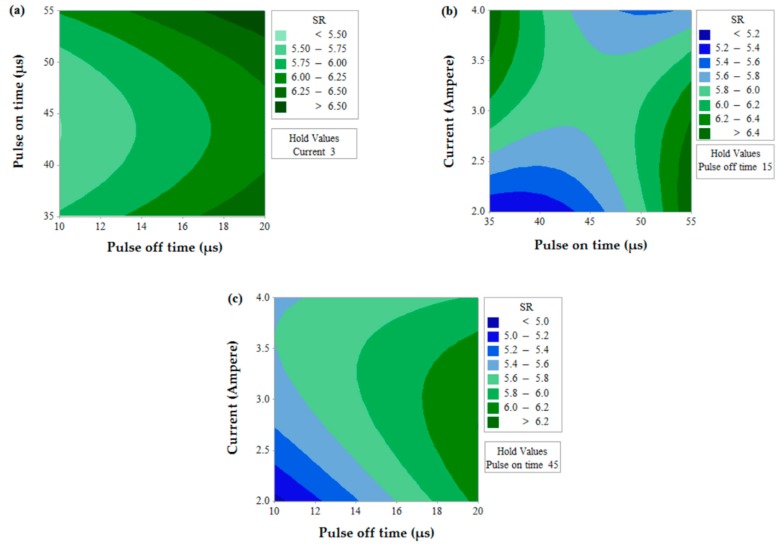
Contour plot for SR.

**Figure 8:**
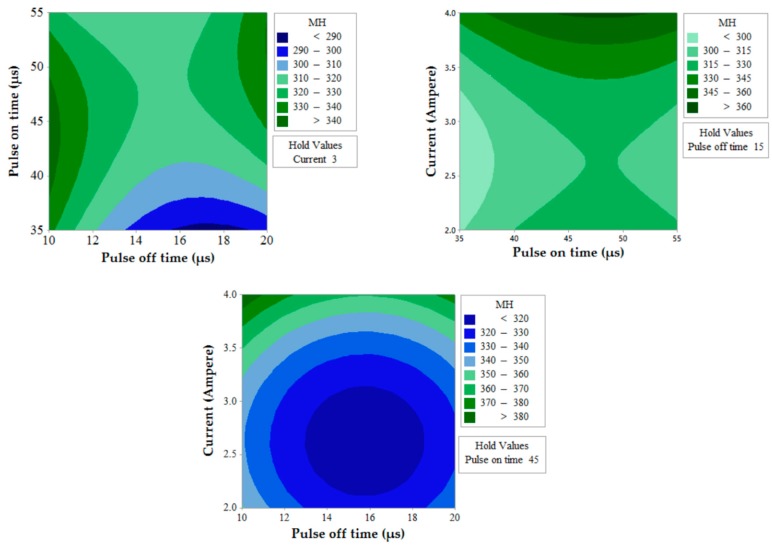
Contour plot for MH.

The prediction of two simultaneous factors (when the third value is held constant) on a single output response is shown in Figure 6 for MRR. A higher value of MRR (>1.05 mm^3^/s) can be obtained when T_off_ value is near to 10 µs and for any value of T_on_ (between 35 µs to 55 µs). The effects of T_on_ and T_off_ also predict that MRR value increases with a decrease in T_off_. Similarly, MRR increases with the decrease in T_off_ value and increase in current for the effect of contour for current and T_off_. This is due to the fact that the number of spark decreases with an increase in pulse off time which thereby lowers MRR and discharge energy increase with an increase in current which thereby increases MRR [35,36]. The effect of T_on_ and current predicts the higher value of MRR when the current is in between 3.2 to 4 A and for any value of T_on_ (between 35 µs to 55 µs).Figure 7 shows the contour plots for SR with the variation of two alternative input variables keeping the third variable as constant. Effect of T_on_ and T_off_ predicts the lower value of SR when T_on_ value is in between 35 µs to 50 µs and T_off_ value is near to 10 µs. A lower value of SR is observed at a lower value of T_on_ and current and maximum SR is obtained at a higher value of T_on_ and current. The discharge of higher pulse energy penetrates into the surface by forming a deep crater and leads to higher SR [20]. Furthermore, a contour plot for current and T_off_ shows the least value of SR when T_off_ varies from 10 to 12 µs and for the current value of 2 A. Figure 8 shows the contour plots for MH. Higher MH is obtained at the three different conditions viz. of T_on_ 35 µs and T_off_ 16 to 19 µs, current near to 4 A and T_on_ 40 µs to 55 µs, and again current 4 A and T_off_ near to 10 µs.

### 3.2. Optimization of Case Studies and Its Validation

**Figure 9:**
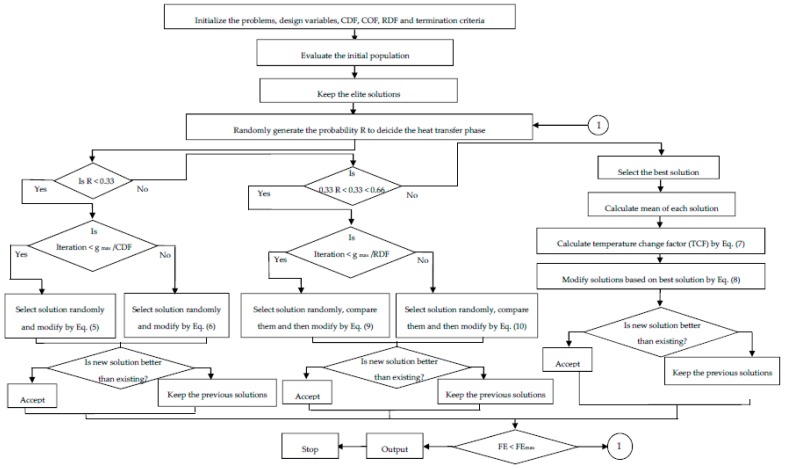
Flow chart of heat-transfer search (HTS) algorithm. Adapted from [31], with permission from © 2017 Elsevier.

The HTS algorithm, proposed by Patel and Savsani [37] works on heat transfer due to the interaction between the system molecules as well as with the surroundings to reach thermal equilibrium. A thermodynamically imbalanced system always tries to achieve thermal equilibrium by heat transfer between the system and its surroundings. The modes of heat transfer are conduction, convection, and radiation plays a major role in setting thermal equilibrium. Thus, the HTS algorithm considers ‘the conduction phase,’ ’the convection phase, ’ and ‘the radiation phase’ to reach an equilibrium state. In the HTS algorithm, all three modes of heat transfer have an equal chance to transfer heat, and one of the heat transfer modes is decided randomly for each generation. The HTS algorithm initiates with a randomly generated population, where the system has ‘n’ number of molecules (i.e., population size) and the temperature level (i.e., design variables). In the next stage, the population is updated in each generation by one of the randomly selected heat transfer modes. Moreover, the updated solution in the HTS algorithm is accepted if it has a better functional value. Subsequently, the worst solutions of the population are replaced by elite solutions. The entire working process of the HTS algorithm is presented in the form of flow chart as shown in Figure 9.

#### 3.2.1. Conduction Phase

The solutions are updated in the conduction phase as per the below Equations (5) and (6),

(5) Xj,i′={Xk,i+(−R2Xk,i), iff(Xj)>f(Xk)Xj,i+(−R2Xj,i), iff(Xj)<f(Xk);If g≤gmaxCDF

(6) Xj,i′={Xk,i+(−riXk,i), iff(Xj)>f(Xk)Xj,i+(−riXj,i), iff(Xj)<f(Xk);If g>gmaxCDF

where, $X_{j,i}^{\prime }$ is the updated solution; j = 1,2,…,n; k is a randomly selected solution; j ≠ k; k ∈ (1,2,…,n); i is a randomly selected design variable; i ∈ (1,2,…,m); g_max_ is the maximum number of generation specified; CDF is the conduction factor; R is the probability variable; R ∈ {0, 0.3333}; r_i_ ∈ {0, 1} is a uniformly distributed random number.

#### 3.2.2. Convection Phase

The solutions are updated in the convection phase as per the below Equations (7) and (8),

(7) $$X_{j,i}^{\prime }=X_{j,i}+R\times \left(X_{s}-X_{\mathrm{ms}}\times \mathrm{TCF}\right)$$

(8) TCF={abs(R−ri), If g≤gmaxCOFround(1+ri), If g>gmaxCOF

where, $X_{j,i}^{\prime }$ is the updated solution; j = 1,2,…,n; i = 1,2,…,m.COF is the convection factor; R is the probability variable; R ∈ {0.6666, 1}; r_i_ ∈ {0, 1} is a uniformly distributed random number; X_s_ be the temperature of the surrounding and X_ms_ be the mean temperature of the system; TCF is a temperature change factor.

#### 3.2.3. Radiation Phase

The solutions are updated in the radiation phase as per the below Equations (9) and (10),

(9) Xj,i′={Xj,i+R×(Xk,i−Xj,i), iff(Xj)>f(Xk)Xj,i+R×(Xj,i−Xk,i), iff(Xj)<f(Xk);If g≤gmaxRDF

(10) Xj,i′={Xj,i+ri×(Xk,i−Xj,i), iff(Xj)>f(Xk)Xj,i+ri×(Xj,i−Xk,i), iff(Xj)<f(Xk);If g>gmaxRDF

where, $X_{j,i}^{\prime }$ is the updated solution; j = 1,2,…,n; i = 1,2,…,m; j ≠ k; k ∈ (1,2,…,n) and k is a randomly selected molecules; RDF is the radiation factor; R is the probability variable; R ∈ {0.3333, 0.6666}; r_i_ ∈ {0, 1} is a uniformly distributed random number.

For each of the objective function considered in different case studies, the HTS algorithm was executed with 10000 function evaluation.

The machining range considered in the HTS algorithm for all case studies is as follows:

For Pulse on time:    1 µs ≤ Pulse on time ≥ 110 µs
For Pulse off time:    1 µs ≤ Pulse off time ≥ 32 µs
For Current:              1 A ≤ Current ≥ 6 A

##### Case: I Optimization of Microhardness (MH)

Machining of shape memory alloys could vastly extend their applications without the deterioration of shape memory effect. MH value is one of the available measures to check the shape memory effect. Lotfi Neyestanak and Daneshmand [32] reported that a larger value of MH shall indicate the continuation of shape memory effect even after machining. In the present case, Equation (11) shows a single objective function, for which optimization was carried out using the HTS algorithm.

(11) $$\mathrm{Obj}\;\left(v_{1}\right)=\left(\mathrm{MH}\right)$$

The maximum attainable value was found to be 870.86 HV from the optimization results. For this maximum value of MH, corresponding input parameters of a pulse on time of 63 µs, pulse off time of 32 µs and current of 6 A was observed. The other output responses were also predicted for the present optimal condition and the validation test was also carried out at predicted input parameters. As the objective of the present case was to obtain maximum MH, other output response variables were not at their optimal values. The experimental validation result of 861.7 HV was observed for MH. The developed model along with HTS algorithm was found to be capable of predicting and optimizing the process parameters as a close relation can be seen between the predicted and measured value. Furthermore, wherever shape memory effect is of prime most requirements with lesser importance to MRR and SR, this present case could become highly useful for industrial applications.

##### Case: II Optimization of MH and Material Removal Rate (MRR)

MRR plays an important role in increasing productivity and thereby making the machining process cost-effective for industries. Without losing the shape memory effect, higher MRR is always preferable for any kind of machining process. Hence in the present case, MH and MRR are combined with different weights by formulating the objective function (shown in Equation (12)) and the HTS algorithm was used to analyze this multi-objective optimization problem.

(12) $$\mathrm{Obj}\;\left(v_{2}\right)=w_{1}\cdot \left(\mathrm{MH}\right)+w_{2}\cdot \left(\mathrm{MRR}\right)$$

As already discussed, higher MH could maintain the shape memory effect, slightly higher weight of 0.6 was assigned to MH and 0.4 for MRR to achieve a higher value of MRR. The optimal value of the objective function for the input variables wasa pulse on time of 28 µs, pulse off time of 5 µs and current of 6 A observed. A confirmation trial was conducted on the obtained set of input parameters (pulse on time of 28 µs, pulse off time of 5 µs and current of 6 A) and are shown in Table 5. The developed model along with HTS algorithm was found to be capable of predicting and optimizing the process parameters as the close relation can be seen between the predicted and measured value as shown in Table 5.

##### Case: III Optimization of MH and Surface Roughness (SR)

Apart from MH, SR of the machined surface becomes an important parameter which denotes the machining quality for certain application requiring aesthetic features or the mating of parts. In the present case, multi-objective optimization was conducted for simultaneous optimization of MH and SR by considering a single objective function wherein suitable weights were given to both responses. MH is considered as higher-the-better and SR as lower-the-better, which are typically a measure of a higher quality of machined surface. Equation (13) shows the objective function which was optimized using the HTS algorithm.

(13) $$\mathrm{Obj}\;\left(v_{3}\right)=w_{1}\cdot \left(\mathrm{MH}\right)+w_{2}\cdot \left(\mathrm{SR}\right)$$

Equal weights of 0.5 were assigned to both the output parameters. Corresponding input parameters of a pulse on time of 65 µs, pulse off time of 32 µs and current of 6 A were observed to give the optimal value of the present objective function. Output responses were mentioned in Table 6 which shows a negligible error between the predicted values and the measured value. This shows that the HTS algorithm performs fairly well in getting required results for multi-objective optimization of the WEDM process.

##### Case: IV Simultaneous Optimization of MH, SR, and MRR

In the present case, a single objective function was formulated by combining all the output responses of MH, SR, and MRR by weights of 0.5, 0.3 and 0.2 respectively. Weights were assigned to output responses by considering their importance for shape memory alloys. In the present objective function (as shown in Equation (14)), MH and MRR are considered as higher-the better whereas SR as lower-the better performance characteristics. Further, the HTS algorithm was used to conduct the multi-objective optimization.

(14) $$\mathrm{Obj}\;\left(v_{4}\right)=w_{1}\cdot \left(\mathrm{MH}\right)+w_{2}\cdot \left(\mathrm{SR}\right)+w_{3}\cdot \left(\mathrm{MRR}\right)$$

Corresponding input parameters of pulse on time of 65 µs, pulse off time of 32 µs and current of 6 A were observed to give the optimal value of the present objective function. Input process parameters obtained for the present case was found to be the same as that of case III. Hence, the similar validation results were used for the present case and were mentioned in Table 6 which shows a negligible error between the predicted values and the measured value. This shows that the HTS algorithm performs fairly well in obtaining the required results for multi-objective optimization of the WEDM process. This shows that the developed models and the combined approach can be useful to predict the accurate and robust results.

### 3.3. Differential Scanning Calorimetry (DSC) Test

**Figure 10:**
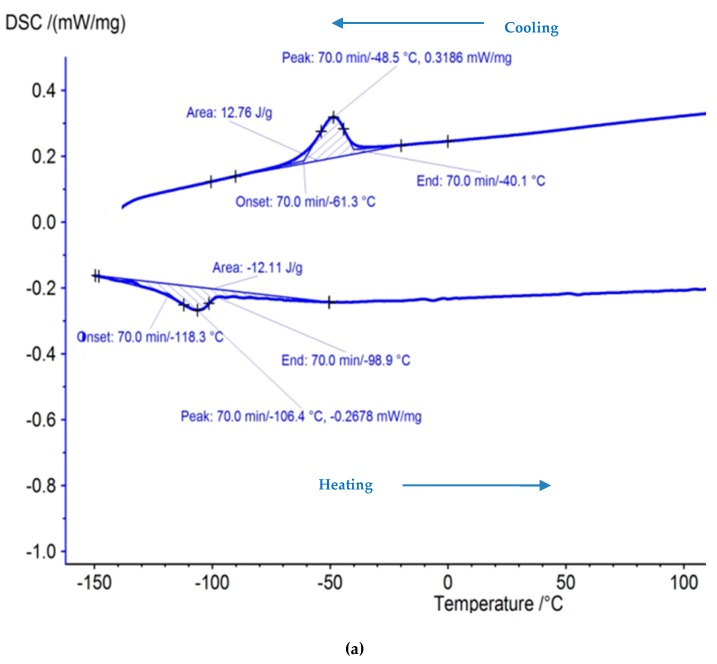

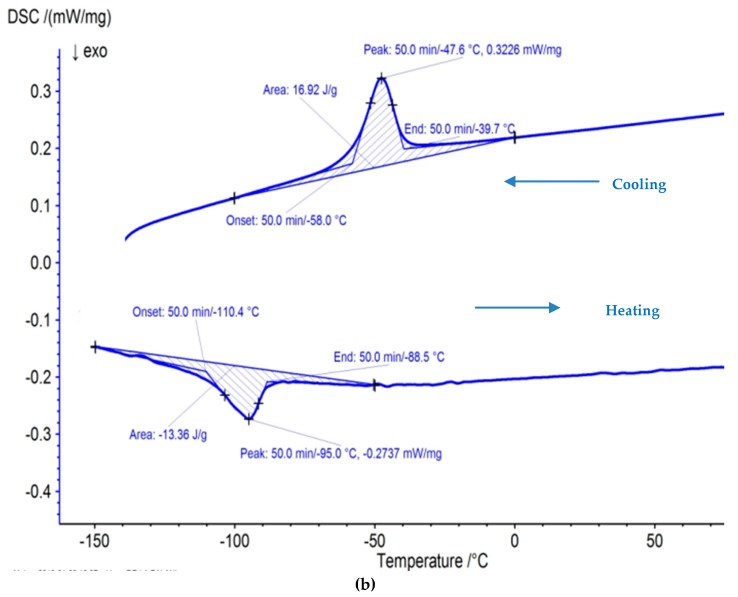
Differential scanning calorimetry (DSC) curve of NiTi alloy for (**a**) unmachined sample (**b**) machined sample at optimized parameter.

A DSC test was carried out to ensure the presence of crucial shape memory effect after machining. This test typically consists of heating and cooling the sample under a controlled environment wherein a plot indicating relationship between temperate and phase change is determined. The machined surface for validation trial of case IV was selected for the DSC test. The austenite start temperature (As) denotes the start of the austenite transformation whereas the austenite finish temperature (Af) represents the end of the transformation. The same type of notation applies for martensite transformation temperatures too (Ms, Mf). Hysteresis is the measure of the difference in transformation temperatures between heating and cooling i.e., |As − Mf|and |Ms − Af| [38,39,40]. The hysteresis and the phase transformation temperatures are mentioned in Table 7. Figure 10a,b shows the DSC curve for the unmachined and machined sample (at final optimized parameters). To determine the transformation temperature, the intersection of a baseline and the tangent to each peak has been considered. DSC curves of the unmachined and machined samples depict the exothermic peak on cooling for the forward transformation from austenite to martensite (B2 to B19′). Furthermore, both samples show endothermic peaks on heating during the reverse transition from martensite to austenite (B19′ to B2). The transformation temperature was observed to be very close to each other for unmachined and machined samples. This shows that the shape memory effect was retained even after machining using the WEDM process. Acceptable change in hysteresis has been observed between unmachined and machined sample values.

### 3.4. Generation of Pareto Optimal Set

Table 8 shows the individual single objective optimization using HTS algorithm for each of the objective (MRR, SR, and MH). It can be observed that when one of the objectives is at its optimal value, the remaining two objectives are away from its maximum/minimum values. For example, when maximization of MRR was carried out, the corresponding values of MH and SR obtained were 423.05HV and 13.70 µm, respectively. However, those values of MH and SR are not clearly at their optimal level. The same can be seen for the remaining two objective function results. Another important conclusion that can be drawn from the table is that, for obtaining the optimum value of each of the objectives individually, the value of input variables are different. These results indicate the conflicting effect of input parameters on the output responses. Such problems can be efficiently tackled by obtaining Pareto optimal points which are basically tradeoffs between output responses.

Simultaneous optimization of two or more than two objectives can be achieved with the help of multi-objective heat transfer search (MOHTS) algorithm which is a multi-objective version of the heat transfer search algorithm [41,42,43]. The non-dominated solutions generated by the MOHTS algorithm are stored in the external archive. Furthermore, ε-dominance based updating method is used to check the domination of solutions kept in the external archive. The Pareto front in MOHTS algorithm was obtained with the help of these non-dominated solutions. The archiving process in the MOHTS algorithm employed a grid-based approach with fixed size archive. The archive stored the best solutions obtained during the execution of the HTS algorithm. Furthermore, the archive is updated in every generation during the execution of the HTS algorithm by adapting the ε-dominance method. The ε-dominance method adopted in the MOHTS algorithm presumes a space having dimensions equal to the number of objectives of the optimization problem. This space further converted into the boxes of ε to ε size by slicing each dimension. Each box holds the solutions generated during the course of optimization. After that, the boxes which are dominated by the other boxes are removed first. Thus, the solutions in those boxes are removed. Afterward, in the remaining box if more than one solution exists then the dominated ones are removed from that box. Therefore, only one solution remains in the box which is non-dominated in nature. Thus, only non-dominated solutions are retained in the archive.

**Figure 11:**
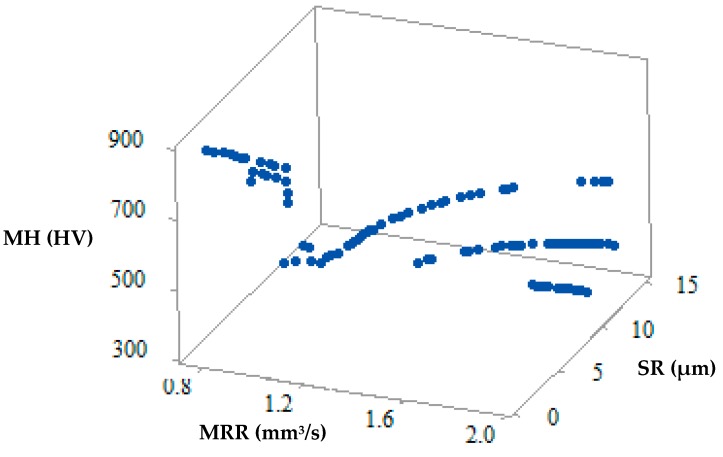
3D Pareto curve of MRR vs. SR vs. MH.

For the simultaneous optimization of all the output responses viz. MRR, SR and MH, the MOHTS algorithm was implemented to obtain the non-dominant Pareto optimal points. Figure 11 shows the Pareto optimal points plotted 3D space. In the 3D plot, MRR is represented on the X-axis, SR on Y-axis and MH on Z-axis. These Pareto optimal points have been obtained at the end of 10,000 evolution functions. Figure 11 shows 100 feasible Pareto points, however, the number of Pareto points can be obtained depending upon the requirement. Each of the point represented on the Pareto curve gives a unique solution and has a corresponding input process parameter. The operator can select any Pareto point and its corresponding input process parameter for machining based on the requirement. In several methods of generating Pareto optimal points, the non-dominant Pareto points have to be identified from the resultant Pareto points. However one of the inherent benefits of using the MOHTS algorithm is the fact that the resultant Pareto points are non-dominant solutions and are obtained in a single step.

**Figure 12:**
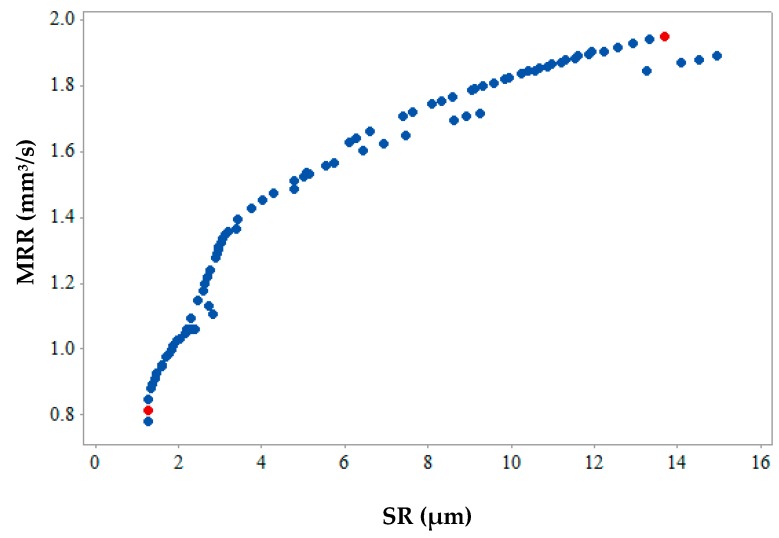
2-D Pareto optimal points for MRR vs SR.

**Figure 13:**
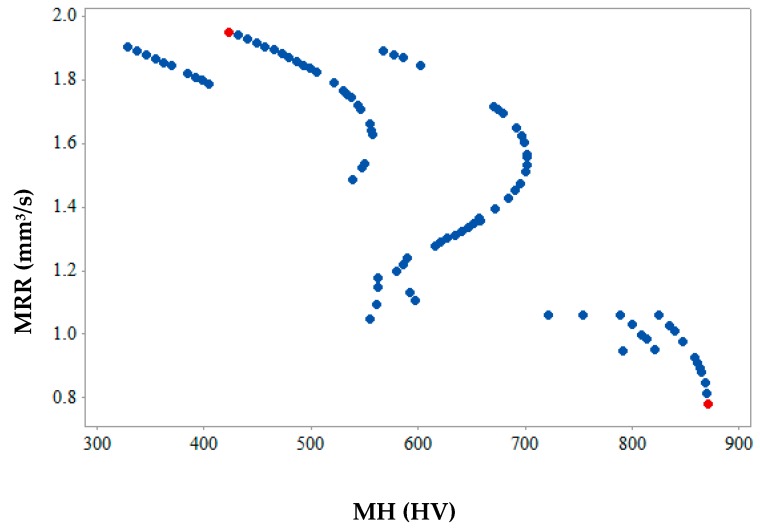
2-D Pareto optimal points for MRR vs. MH.

**Figure 14:**
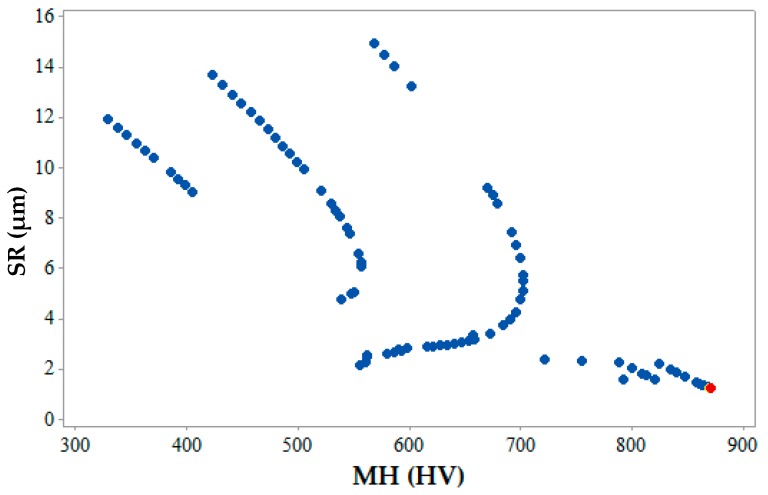
2-D Pareto optimal points for SR vs. MH.

Figure 12, Figure 13 and Figure 14 show the 2-D views of the generated solutions for the better understanding of 3-D Pareto points. The effect of third response parameter is included in all the 2D views as shown in Figure 12, Figure 13 and Figure 14. Over the entire search space, a discrete distribution of Pareto points has been obtained. These points give a clear picture for better understanding along with the inclusion of the effect from the third response parameter. Complex dependencies and highly conflicting effects can be evidently seen from 2D views of input variables on output responses. In existing literature, simultaneous optimization of two output responses has been reported completely neglecting the third output response [33], incorporated in the present study. This gives a better idea to designers and manufacturers on the selection of input parameters for the WEDM process for SMA. Figure 12 shows the maximum and values of MRR and SR are 1.95 mm^3^/s and 1.28 µm (as shown by red points) respectively. This shows that when maximum MRR needs to be achieved for higher production rate, the SR values are also at higher side showing the conflicting occurrence. Hence a Pareto point with its corresponding input parameters will be selected which would be a trade-off between these two values. A similar conclusion can be drawn from Figure 13 for the 2D view of MRR vs. MH which also shows that when maximum MRR needs to be achieved for higher production rate, the MH values are at lower side showing the conflicting occurrence. Whereas when maximum MH is considered as an output, MRR value is at a minimum. The maximum and minimum values of MRR and MH are 1.95 mm^3^/s and 870.86 HV (as shown by red points), respectively. Figure 14 gives the maximum and minimum values of SR and MH as 1.28 µm and 870.86 HV (as shown by red and yellow points) respectively for the 2D view of SR vs. MH. For some Pareto points, when SR is at its peak value, corresponding values of MH are not at its peak. This might be due to the effect of the third response parameter evident on the generated 2-D graphs. The selection of input and output variable values in the actual WEDM process is very complex, which requires true dependencies for the decision of input process parameter selection. In the current study, MH value becomes more important considering the fact that the possession of a shape memory effect is a must after machining.

## 4. Conclusions

In the current study, desirable results for the parametric optimization of superelastic SMAs during the WEDM process have been achieved. The following important conclusions can be drawn from the study:

- The regression models generated for the selected output response variables were found to be robust, verified using ANOVA. Residual plot analysis confirmed the prediction capability of the generated models of MRR, SR and MH.
- T_off_ and current were found to be most significant parameters influencing SR and MH respectively, where as T_off_ and current were significantly influencing MRR. Contour plots analyses were used to identify the significance of input variables on the individual output responses.
- The heat-transfer search (HTS) algorithm was found effective in predicting and optimizing the input values for four different case studies under consideration. The same was confirmed using validation tests. A close correlation between predicted and achieved values was obtained.
- DSC tests carried out for case IV indicated negligible difference between A_f_ temperature for the machined and unmachined surface which confirmed the retention of shape memory effect even after WEDM. This can be considered as one of the most notable outcomes of the study.
- 3-D Pareto curves were generated which effectively presented the solution for the simultaneous optimization of three output responses such as MRR, SR, and MH. A multi-objective version of HTS termed the MOHTS algorithm was successfully implemented for this.
- The complex relationship and conflicting nature of between input parameters, their interactions and output responses was evident from the scattered nature of the 2-D Pareto fronts.

## Figures and Tables

**Table 1:** Chemical composition (wt.%) of Nitinol.

**Element**	Ti	Ni	Co	Cu	Cr	Fe	Nb	C	H	O	N
**Content (wt.%)**	Balance	55.78	0.005	0.005	0.005	0.012	0.005	0.04	0.001	0.035	0.001

**Table 2:** Process parameters and their levels.

Factors	Process Parameters	Level 1	Level 2	Level 3
A	Pulse on time (T_on_), µs	35	45	55
B	Pulse off time (T_off_), µs	10	15	20
C	Discharge current, Ampere	2	3	4

**Table 3:** Process parameters and their levels.

Run	Pulse on Time (µs)	Pulse off Time (µs)	Current (Ampere)	MRR (mm^3^/s)	SR (µm)	Microhardness (HV)
1	35	10	3	1.230948122	5.637	330.2
2	55	10	3	1.065245598	5.986	342.3
3	35	20	3	0.714888337	6.453	275.2
4	55	20	3	0.756738988	6.82	342.8
5	35	15	2	0.675983558	5.322	308.6
6	55	15	2	0.666859456	6.58	301
7	35	15	4	1.066357739	6.595	346.5
8	55	15	4	1.103461538	5.577	351.3
9	45	10	2	0.845274725	4.944	341.7
10	45	20	2	0.541463415	5.925	354.2
11	45	10	4	1.23034188	5.638	383.9
12	45	20	4	0.874333587	5.762	374.6
13	45	15	3	0.92275641	6.053	326.3
14	45	15	3	0.925	5.484	309.5
15	45	15	3	0.921634615	6.098	316.3

**Table 4:** Analysis of variance (ANOVA) for MRR, SR and microhardness.

**ANOVA for MRR**					
**Source**	**SS**	**MS**	**F**	**P**	**Significance**
**T_on_**	0.001149	0.001149	1.07	0.328	-
**T_off_**	0.275425	0.275425	256.99	0.000	Significant
**Current**	0.298345	0.298345	278.37	0.000	Significant
R–Sq = 98.41 %, R–Sq (Adj) = 97.53%					
**ANOVA for SR**					
**Source**	**SS**	**MS**	**F**	**P**	**Significance**
**T_on_**	0.11424	0.11424	2.51	0.157	-
**T_off_**	0.94875	0.94875	20.85	0.003	Significant
**Current**	0.08020	0.08020	1.76	0.226	-
R–Sq = 91.71 %, R–Sq (Adj) = 83.41%					
**ANOVA for MH**					
**Source**	**SS**	**MS**	**F**	**P**	**Significance**
**T_on_**	739.2	739.2	3.14	0.120	-
**T_off_**	329.0	329.0	1.40	0.276	-
**Current**	2842.6	2842.6	12.07	0.010	Significant
R–Sq = 85.52 %, R–Sq (Adj) = 71.03%					

**Table 5:** Validation results for case study II.

Condition	MRR (mm^3^/s)	SR (µm)	Microhardness (HV)
Predicted by HTS Algorithm	1.6938	8.62	679.05
Experimentally measured values	1.5921	8.89	654.32
% ERROR	6	3.13	3.64

**Table 6:** Validation results for case studies III and IV.

Condition	MRR (mm^3^/s)	SR (µm)	Microhardness (HV)
Predicted by HTS Algorithm	0.81324	1.28	870.21
Experimentally measured values	0.77271	1.35	855.55
% ERROR	4.98	5.46	1.68

**Table 7:** Phase transformation temperatures and hysteresis.

Nitinol Sample	A_s_ (°C)	A_f_ (°C)	M_s_ (°C)	M_f_ (°C)	Hysteresis, |A_s_ − M_f_| (°C)
Unmachined	−58	−39.7	−88.5	−110.4	52.4
Machined	−61.3	−40.1	−98.9	−118.3	57

**Table 8:** Results of single objective optimization.

Objective Function	Design Variables			Objective Function Value		
	Pulse on Time	Pulse off Time	Current	MRR (mm^3^/s)	MH (HV)	SR (µm)
Maximum MRR	10	5	5	**1.9503**	423.05	13.70
Maximum MH	63	32	6	0.7803	**870.86**	1.29
Minimum SR	65	32	6	0.8132	870.21	**1.28**

## Nomenclature

A	Ampere
A_f_	Austenitic start
A_f_	Austenitic finish
ANOVA	Analysis of variance
BBD	Box-Behnken design
DSC	Differential scanning calorimetry
HTS	Heat transfer search
IP	Peak current (Ampere)
M_f_	Martensitic start
M_s_	Martensitic finish
MH	Microhardness (HV)
MOHTS	Multi-objective heat transfer search
MRR	Material removal rate (mm^3^/s)
RSM	Response surface methodology
SMA	Shape memory alloy
SMAs	Shape memory alloys
SR	Surface roughness (µm)
SV	Spark gap voltage
T_on_	Pulse on time (µs)
T_off_	Pulse off time (µs)
WEDM	Wire electric discharge machine
